# Perception of transplant recipients and professionals about health care following kidney transplantation

**DOI:** 10.1590/1980-220X-REEUSP-2024-0237en

**Published:** 2025-02-14

**Authors:** Sâmia Jucá Pinheiro, Carla Daniara Feitosa Coelho, Jean Augusto da Silva, Cristefânia Meirú de Lima, Annaíza Freitas Lopes de Araújo, Klarissa Karine Lima Maracaipe, Dayani Galato

**Affiliations:** 1 Empresa Brasileira de Serviços Hospitalares, Hospital Universitário Walter Cantídio, Fortaleza, CE, Brazil.; 2 Universidade de Brasília, Faculdade de Ceilândia, Brasília, DF, Brazil.; 3 Empresa Brasileira de Serviços Hospitalares, Hospital Universitário de Brasília, Brasília, DF, Brazil.

**Keywords:** Kidney Transplantation, Postoperative Care, Comprehensive Health Care, Health Communication, Trasplante de Riñón, Cuidados Posoperatorios, Atención Integral de Salud, Comunicación en Salud

## Abstract

**Objective:**

To explore the perception of renal transplant recipients and professionals about health care following kidney transplantation.

**Method:**

Exploratory, qualitative study, carried out through focus groups and questionnaire application, in 2023, with kidney transplant recipients and healthcare professionals. The analysis was performed with the support of the software IRaMuTeQ^®^.

**Results:**

Twenty-four transplant recipients and 18 professionals participated. From data processing, two thematic groups emerged: use of immunosuppressive medications and dietary habits after kidney transplantation, and other non-pharmacological care and health monitoring after transplantation.

**Conclusion:**

The major concern of transplant recipients is related to the use of medication and eating habits; and professionals consider the use of medication and health monitoring extremely important for the proper functioning of the transplanted graft. However, there is an importance of non-pharmacological care, for both groups, in the face of adequate therapeutic follow-up.

## RESUMEN

**Objetivo:**

Explorar las percepciones de los profesionales y los receptores de trasplantes de riñón con respecto a la atención médica después del trasplante.

**Método:**

Estudio exploratorio, cualitativo, realizado mediante grupos focales y aplicación de cuestionarios, en el año 2023, con receptores de trasplante renal y profesionales de la salud. El análisis se realizó con el apoyo del software IRaMuTeQ^®^.

**Resultados:**

Participaron 24 trasplantados y 18 profesionales. Del procesamiento de datos surgieron dos grupos temáticos: uso de medicamentos inmunosupresores y hábitos alimentarios después del trasplante de riñón, y otros cuidados no farmacológicos y seguimiento de la salud postrasplante.

**Conclusión:**

La mayor preocupación de las personas trasplantadas está relacionada con el uso de medicamentos y hábitos alimentarios; y los profesionales consideran de suma importancia el uso de medicamentos y el seguimiento de la salud para el buen funcionamiento del injerto trasplantado. Sin embargo, existe importancia de la atención no farmacológica, para ambos grupos, frente a un adecuado seguimiento terapéutico.

## INTRODUCTION

Kidney transplantation is considered a renal replacement therapy for patients with advanced chronic kidney disease (CKD), providing better survival compared to hemodialysis^(1)^. However, long-term success depends not only on the medical-surgical procedures, but also on the quality of care received before and after these procedures are performed^(2)^. The way transplant recipients and their families perceive and engage with this care can significantly impact treatment adherence and, consequently, outcomes, whether clinical or humanistic^(3)^.

Individuals who have undergone a kidney transplant face a complex pre-transplant process, which involves undergoing several exams and consultations to make them eligible to enter the waiting list for the organ. After the procedure, they must strictly adhere to a regimen of immunosuppressive medications, undergo regular follow-ups with the healthcare team, and adopt lifestyle changes to prevent infections and minimize the risk of transplanted organ rejection.

This ongoing care burden can be challenging for patients, affecting their physical and emotional well-being, but also for healthcare professionals and institutions, given the logistics to be executed and the costs involved in achieving the goal^(4)^.

Healthcare professionals also play a key role in providing pre- and post-kidney transplant care. Technical-scientific knowledge about treatment protocols, about the difficulties faced by these people in the face of the complexity of managing self-care, and about strategies to improve adherence, is essential to shape care practices. However, these professionals also face challenges often related to high workload and reduced ability to understand the needs of each transplant patient, which is essential to promoting personalized care^(5)^.

The bond and mutual collaboration between health professionals and patients in the face of these therapeutic multicomponents are vital components for the long-term success of kidney transplant recipients, where the importance of exploring mutual perspectives and experiences in the context of post-transplant care gains considerable prominence^(6)^.

Given the complexity of post-transplant care, it is believed that qualifying the perception of kidney transplant recipients and health professionals about this care can play a crucial role in effective health management and self-care with the aim of improving clinical results and the experience of these people through adaptation strategies for better adherence to treatment.

Therefore, the study aims at exploring the perception of renal transplant recipients and professionals about health care following kidney transplantation.

## METHODOLOGY

### Design of Study

This is an exploratory, descriptive study, presenting a qualitative approach through Focus Group (FG) and application of questionnaires.

### Population, Local and Selection Criteria

The study was carried out at the kidney transplant outpatient clinic, in August 2023, with 24 people who underwent kidney transplantation and 18 health professionals working in the transplant unit, from August to December 2023, all in a reference hospital in the Federal District.

### Sample Definition

Patients who had undergone kidney transplants and were treated at the post-transplant clinic, who were at least 18 years old, were included, and those who were lethargic at the time of collection were excluded. People were invited to participate in the research while they were waiting for care at the outpatient clinic.

The health professionals included in the study met the following criteria: having worked in the transplant unit for at least one year, and having specialization and/or residency in transplantation, regardless of the length of time they had worked. The exclusion criterion was being on vacation or leave.

### Data Collection

Two FGs were carried out, with 12 participants, with transplant recipients: through a specific script with questions and aspects related to health care guidelines after kidney transplantation and issues of interest suggested by participants during the groups, on different days, which lasted approximately 90 minutes each. Participants were invited while they were waiting for their appointment at the kidney transplant clinic on the days the groups were held. Everyone in the waiting room accepted the invitation and no one withdrew from participating during the activity. The group was led by a professional and had three rapporteurs. The meetings were audio recorded.

Information regarding health professionals was collected through the application of a questionnaire prepared by the authors and applied in-person, containing questions about professional category, qualifications and length of service, and an open question about essential care for patients after kidney transplantation.

### Data Analysis and Treatment

The patients’ sociodemographic and clinical data and professional issues were subjected to descriptive analysis. The data originating from the aspects addressed around the theme in the FG, obtained through the transcription of the audios and the reporting notes, and those originating from the professionals’ responses were entered into a database and processed in the software *Interface de R pour Analyses Multidimensionnelles de Textes et de Questionneires* (IRaMuTeQ^®^), version ٠.٧ Alpha٢, with a lexical analysis performed using Similarity Analysis and Descending Hierarchical Classification.

The IRaMuTeQ^®^ combines advanced statistical methods, making it accessible to researchers with different levels of experience in data analysis. Similarity analysis is a technique for analyzing textual data, providing a visual and semantic understanding of textual content, and allowing researchers to explore the structure and content of texts in a comprehensive way^(7,8)^. The Descending Hierarchical Classification is a technique that allows grouping similar textual elements to identify semantic structures in large volumes of text. In IRaMuTeQ^®^, the chi-square test (X^2^) is a statistical tool used to analyze the association between words and categories in a *corpus* of text, helping to build a dendrogram that represents the classes with their most characteristic words^(9)^).

### Ethical Aspects

The study was the result of two stages of doctoral research and was analyzed and approved by the Research Ethics Committee of the School of Ceilândia, with opinion no. 67087723.2.0000.8093, in accordance with Resolution no. 466, of December 12, 2012, of the National Research Ethics Council. Participants were informed that participation was voluntary and it was highlighted that the refusal would not cause any harm to the care provided to the patients, nor would there be any disagreement from the researchers regarding the probable refusal of the professionals. Everyone signed the consent form beforehand.

To preserve anonymity, patients were characterized by the letter P and their identification number, followed by the letter G and the number corresponding to the order in which the FG was performed, for example: P1G1 (patient one of the first FG performed). Professionals were characterized by the letter E and their identification number, such as E1 (professional one). The study was prepared following the recommendations for developing qualitative research from COREQ (Consolidated Criteria for Reporting Qualitative Research).

## RESULTS

Twenty-four transplant recipients and 18 professionals participated in the study. Initially, the characterization data of people undergoing transplantation will be presented (Table 1).

**Table 1:** Sociodemographic and clinical profile of transplant recipients who participated in the FG on the perception of care after kidney transplantation – Brasilia, DF, Brazil, 2023.

Kidney transplant patients	N	%
**Sex**		
Female	12	50
Male	12	50
**Race**		
Black	1	4%
Brown	21	88%
Yellow	0	0
White	2	8%
**Marital status**		
Single	3	12%
Married	5	21%
Common law marriage	11	46%
Divorced	4	17%
Widower	1	4%
**Level of Education**		
No schooling	1	4%
Elementary education	8	33.5%
High school	7	29.5%
Higher education	6	25%
Postgraduate studies	2	8%
**Age**		
Adults	16	66.5%
Elderly	8	33.5%
**Causes of CKD**		
Unknown	16	66.5%
Autosomal dominant polycystic kidney disease	3	12.5%
Diabetes Mellitus	2	8%
IgA nephropathy	1	4%
Crescentic glomerulonephritis	1	4%
Antibiotic therapy nephrotoxicity	1	4%
**Transplant time (years)**		
<1	10	41.5%
1–5	9	37.5%
Above 5	5	21%
**Donor type**		
Alive	5	21%
Deceased	19	79%
**Retransplantation**		
Yes	4	17%
No	20	83%
**Total**	**24**	**100%**

The transplanted people were evenly distributed in relation to sex. The majority considered themselves brown; the majority was married or in a common-law marriage, corresponding to 67%. Regarding age, 66.5% were adults and 33.5% were older people. The majority was unaware of the cause of CKD, corresponding to 66.5%.

The participating professionals presented diverse categories, with the largest number being represented by doctors and nurses, both with four and eight, respectively; two physiotherapists; one pharmacist; one nutritionist; one social worker; and one psychologist. The majority (13) had a specialist or residency title; four had a master’s degree and one had a doctorate degree. Nine had less than five years of experience in the kidney transplant service, and only one had more than ten years of experience.

**Figure 1:**
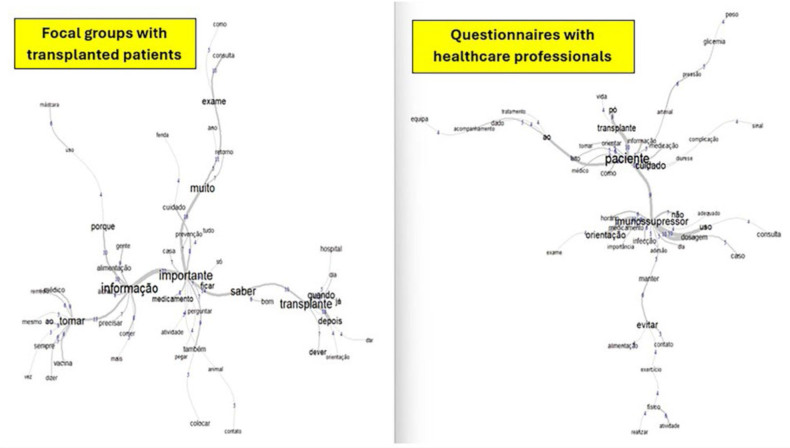
Similarity analysis corresponding to the results of the focus groups with patients and the questionnaires with professionals about health care after kidney transplantation.

The similarity analysis of the texts obtained through the FG with the transplanted people and the questionnaires with the health professionals who worked in the assistance allowed the elaboration of Figure 1. In the images, it is possible to observe the synthesis of words that allow the understanding of the object of the study.

Among the words that stood out in the FG, four axes organized in a transversal manner were observed. They are: *transplant (transplante), knowledge (saber), important (importante), and information (informação)*. The word *guidance* (*orientação*) appears as a branch on the axis of the *transplant*, which is directly connected to the axis of the *knowledge*. On the axis of words *information* and *important* branches appear related to *food* (*alimentação*) and to the *medicines (medicamentos)*; and a more horizontal branch related to the word *take (tomar)*, being linked to the use of *medicines* and *vaccines (vacinas)*.

The graphic representation of the words evoked by the patients demonstrates the understanding related to what is important after the transplant, as well as the need for guidance and information concerning therapeutic follow-up, especially regarding aspects related to medication and nutrition, as can be evidenced by the transcription of the statements of some participants:

P1G1: *At first I thought I was going to die from all the medication I had to take and I didn’t understand anything. I was told that I shouldn’t eat a lot of red meat. I’m lost not knowing what to eat.*

P2G1: *When I was discharged, the nutritionist gave general advice, which was very superficial. The issue of food, what works for me may not work for someone else. When the test results come out, we don’t know what to eat. Another patient, when she was discharged with me, didn’t know what to eat.*

P3G1: *It would be interesting to have guidance regarding the application of insulin, I was very afraid of applying it alone when I started using it, 4 years after the transplant. If there was a video, a picture... explaining how to apply it, it would have been better.*

P9G1: *The diet raises doubts, at discharge they deliver it very superficially. The relationship between diet and test results is important. I already notice when I eat something wrong, I already notice changes in my body... I feel dizzy, I have a headache... and when the test results come back I already know that I exagerated.*

P1G2: *The main information is about food, you have to know what you can and cannot eat to avoid losing your kidney. In hemodialysis we knew what to do. Medication is also very important... the right medication at the right time, otherwise there will be rejection.*

P2G2: *Information on how to take the medications... times, doses... I thought it was great to receive the medication table upon discharge, explaining exactly how to use prednisone.*

P3G2: *The greatest care you need to take is with food and water. I don’t drink water from just anywhere. In hemodialysis, if you overeat, the machine takes it away... with a transplant, that doesn’t happen.*

P4G2: *Food, medication, and physical activity are the most important information that I think you should have.*

Now with the data relating to the multidisciplinary team, the word *patient* appears more centered in text *corpus*. From it, the words *transplant* and *care (cuidado)* branch closer, and the word *immunosuppressant* (*imunossupressor*) is characterized in an evident way, followed by the word *avoid (evitar),* which appears in a branch further down. *Team (equipe), follow-up (acompanhamento), treatment (tratamento), information (informação),* and *complication (complication)* appear closer linked to the word *patient (paciente)*; and *schedule (horário), dosage (dosagem), guidance (orientação), importance (importância),* and *infection (infecção)*, for example, appear more related to the word *immunosuppressant*. The graphic representation also highlights the concern of health professionals about what the patient should avoid for better development of the treatment, where the relationship between the word *avoid* with *food, exercise, activity* and *contact* is observed.

The emphasis on the words *immunosuppressant* and *guidance* is quite evident in the participants’ reports, in which the majority highlighted the correct use of medications as the first guidance to be provided post-transplant, and can be observed in several reports, some of which are represented below:

E2: *The main guidelines are: Take your medication at the correct times, do not miss any doses; take immunosuppressants away fro*m meals.

E3: Some of the essential measures include: Immunosuppressant care – strict adherence to immunosuppressive therapy. To prevent transplanted organ rejection, the patient must take the medication as prescribed by the doctor, following the times and doses indicated.

E10: *During hospital discharge, patients need to be advised about the risks of losing the transplant resulting from the inappropriate use of immunosuppressants.*

Despite the striking evidence surrounding immunosuppression, there is also concern about non-pharmacological care, in which an important link stands out in the graphic demonstration between *patient* and *care*, inserted in the same zone of *transplant,* and with important ramification with the words *information*, *follow-up* and *treatment*, which can be highlighted through some of the reports:

E1: *Essential care in the late post-operative period – after three or four months of surgery, physical exercises in the gym are allowed, as a form of training and physical conditioning. To do this, you must talk to your doctor and request permission. Several benefits are observed with the practice of regular physical activity, among them: prevention of osteoporosis, diabetes and hypertension; gain in muscle mass; improvement in the quality of sleep and mood.*

E12: *Adherence to drug and non-drug treatment (healthy lifestyle habits for successful treatment - diet, physical activity, and regular follow-up with the healthcare team).*

E13: *Use of masks, sunscreen. Avoid crowds, especially in the first six months after the transplant. Wash your hands before meals and after using the bathroom. Attend appointments and take exams regularly.*

E18: *Health monitoring with a focus on patients’ emotiona*l aspects.

**Figure 2:**
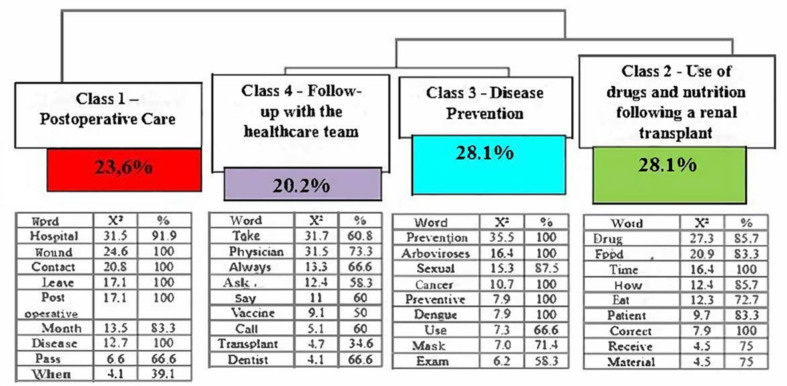
Dendrogram corresponding to the classes found through the processing of the text *corpus* originating from the FG carried out with transplanted people.

In the Descending Hierarchical Classification (Reinert Method), through the processing of data from the FG with patients, 111 text segments (TS) were analyzed with a utilization of 80.18% of the TS, which generated four classes that were named through the idea conveyed by the words found. They are – Class 1 – Post-operative care; Class 2 – Use of medication and nutrition after kidney transplant; Class 3 – Disease prevention; and Class 4 – Follow-up with the health team. Figure 2 shows the dendrogram with the relationship between the classes.

It can be seen that class 1 represented 23.6% of the text segments; classes 2 and 3 represented 28.1% each; and class 4 represented 20.2%. Ten words were included in each class, with the cut-off point for inclusion being a chi-square value (X^2^) greater than or equal to 3.84 and with p < 0.05, being presented in the dendrogram to provide a better understanding when developing the classes.

The classes generated from the Descending Hierarchical Classification through the processing of data obtained from questionnaires with health professionals, and with a 90.67% utilization of TS, were called: Class 1 – Follow-up with the health team; Class 2 – Responsible use of medications; Class 3 – Non-pharmacological care after transplantation; Class 4 – Health monitoring after kidney transplantation.

**Figure 3:**
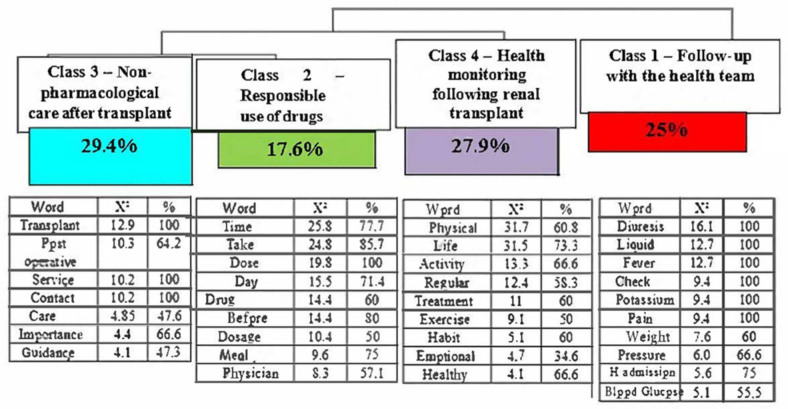
Dendrogram corresponding to the classes found through the processing of the text *corpus* of the questionnaires applied to health professionals who work in providing care to kidney transplant recipients.

Figure 3 shows the classes found through the processing of data from the multidisciplinary team’s questionnaires.

Class 1 represented 25% of TS; 2, 17.6%; class 3 represented 29.4%; and class 4 represented 27.9%. As in the dendrogram related to the text *corpus* of the patients, the words, which also had as a cut-off point the chi-square value (X^2^) greater than or equal to 3.84, and with p < 0.05, were presented in the professionals’ dendrogram to provide a better understanding when preparing the classes.

The classes generated by means of the most frequent words in the text *corpus*, in the two groups analyzed, were extremely valid to guide the discussion of the findings of the present study with other published research, highlighting the most prevalent care needs in patients, as well as emphasizing the direction of care by professionals in the post-transplant period.

## DISCUSSION

In the perception of transplant recipients, information related to diet and medication use was evident, as well as the importance of undergoing tests and attending appointments, in addition to other ramifications related to care for the surgical wound, disease prevention, home care, and contact with animals. In the perception of healthcare professionals involved in care, the patient was placed at the center of care and a greater importance of pharmacological care was demonstrated, mainly related to immunosuppressive drugs. However, there is also an emphasis on non-pharmacological care, demonstrating, through lexical analysis, the importance of eating and exercising for a better quality of life after transplantation.

In this context, through similarity analysis and Reinert’s method, two thematic groups were created to guide the discussion of the data. They are:

### Use of Immunosuppressant Drugs and Dietary Habits After Kidney Transplantation

The importance given to medications by transplant recipients and professionals is understandable, given that the safe administration of immunosuppressants is essential, since these drugs play a role in preventing rejection of the transplanted organ by suppressing the individual’s immune response. The success of the transplant depends on adherence to a prescribed medication regimen, strictly following the doses and times recommended by the health team^(10)^.

Among the most common are tacrolimus, everolimus, sirolimus, mycophenolate sodium and mofetil, cyclosporine, azathioprine, and corticosteroids. These drugs may interact with other medications and foods, as well as cause adverse events that can impact patients’ quality of life^(11)^. Consequently, it is essential to provide detailed information to patients about the correct use of medications, as well as the various problems that may arise, such as increased risk of infections, diabetes, hypertension, dyslipidemia, and cancer^(12,13)^.

In the current study, there are reports of patients who demonstrated concern regarding dietary restrictions, especially those who were undergoing hemodialysis or peritoneal dialysis as replacement therapies before transplantation. The fact is that people who have had kidney transplants have different nutritional recommendations due to the proper functioning of the new kidney. Nevertheless, many patients are still wary of consuming foods that were previously considered prohibited or not recommended during other renal replacement therapies^(14)^.

A balanced diet rich in fruits, vegetables, whole grains, and lean proteins is recommended, while limiting the consumption of sodium, saturated fats, and sugars^(15)^. The assessment of serum levels of sodium, potassium, phosphorus and magnesium, for example, is important to define the course of action between restriction or supplementation if the patient needs it. Adequate fluid intake is also important to maintain hydration and proper kidney function. Proper food hygiene is critical, including washing food thoroughly, refrigerating perishable foods, and avoiding the consumption of potentially contaminated food and/or food prepared in places of unknown or dubious origin^(14)^.

Some food can interact with medications, altering their effectiveness or increasing the risk of side effects. For instance, excessive grapefruit consumption may increase blood levels of certain medications, such as tacrolimus and cyclosporine; just as herbal supplements may interfere with kidney function and/or decrease the effectiveness of immunosuppressants. Therefore, it is essential that patients follow specific guidelines on nutrition provided by the nutritionist from the moment of discharge from hospital^(13)^.

In short, the diet of a kidney transplant recipient must be carefully planned to avoid interactions with medications, especially immunosuppressants, as well as to promote adequate health in the long term, reducing the risks of developing dyslipidemia, diabetes, and high blood pressure, for example^(16)^.

### Other Non-Pharmacological Care and Post-Transplant Health Monitoring

In addition to drug therapy, patients need general care to maintain good health over time. Thus, care for transplant recipients must be holistic and comprehensive. This includes taking care to adopt healthy lifestyle habits, such as physical activity, safe sexual practices, and stress management, as well as practices related to preventing the development of chronic diseases and the onset of infectious diseases, with attendance at consultations and exams with the multidisciplinary team being essential^(17)^.

Sexual activity is permitted six weeks after the transplant or when the person feels ready and is not experiencing pain. However, people should avoid unprotected sex to reduce the risk of infections that could negatively affect the transplanted kidney or cause serious complications. It is advisable to communicate any concerns related to sexual activity to the healthcare team in charge of follow-up, as they can provide personalized guidance on achieving a healthy and safe sex life. Furthermore, it is important for transplanted women to communicate their desire to become pregnant, since pregnancy is only recommended one year after the transplant and, often, there is a change in immunosuppressant medications^(17)^.

The current study, through the results found, highlights the importance of early detection and prevention of diseases among kidney transplant recipients. They should be advised regarding the presence of inflammatory signs in the region of the surgical wound, persistent fever, chills, pain when urinating, increased pain or sensitivity in the region of the transplanted kidney, intense fatigue, malaise, and changes in appetite. These signs may indicate anything from local infections in the surgical wound and urinary tract to serious systemic infections. Rapid identification and treatment of these complications can preserve the function of the transplanted kidney and the individual’s quality of life^(18)^.

Another aspect of care after transplantation is related to guidance regarding immunization of transplant recipients before the transplant, with it being crucial to receive all recommended vaccines, since, after the transplant, vaccines of live and/or attenuated viruses cannot be administered due to the risk of causing an active infection due to the immunological suppression condition of transplant recipients^(19)^.

Passive immunization in transplant recipients is less effective than in immunocompetent individuals, which is why it is essential to vaccinate transplant recipients, mainly due to their greater vulnerability to infections. Individual hesitancy about vaccination, often promoted by anti-vaccine movements, can put people’s health at risk by decreasing community protection against vaccine-preventable diseases. Therefore, it is essential that health professionals inform the general population about the benefits of vaccines, as well as provide guidance on the harm caused by not getting vaccinated^(20,21)^.

It is also essential that transplant recipients be instructed on how to prevent mosquito bites, as well as on seeking immediate medical care if they present symptoms suggestive of arboviroses, such as dengue, zika, and chikungunya, for early management that minimizes the risk of serious complications. Although the course of the disease appears similar to that of the general population, kidney transplant recipients who develop dengue fever, for example, may experience greater complications due to a compromised immune response and the presence of associated comorbidities, which would result in a longer hospital stay^(22)^.

A study published in 2023 about a Benchmarking of educational health technologies to assist in therapeutic adherence of transplant patients highlighted that guidelines related to the importance of carrying out exams and returning for consultations, to aspects related to hygiene and management of post-transplant clinical complications were present in all technologies found, together with responsible use of medicines and healthy eating practices^(23)^. Physical activity practice was also evidenced in most available technologies. Therefore, the patients’ needs and perceptions of professionals’ care highlighted in this study corroborate the aforementioned research.

Physical activity becomes essential after a kidney transplant due to the benefits it provides, such as: improving cardiovascular health, maintaining an adequate weight, reducing the risk of metabolic complications, and promoting muscle strengthening. A study developed in 2023, in the United States, on the development of a lifestyle program among kidney transplant patients, highlights that patient participation was essential to develop healthy behaviors in the program, and revealed that transplant patients showed interest in joining the program with physical activity training and nutritional personalization^(24)^.

To better assist in treatment adherence, patients need ongoing education in transplant treatment. Accordingly, professionals need constant improvement and educational practices to help in this process and, consequently, improve survival and quality of life. This can be demonstrated by a study carried out in a large hospital in Ethiopia with kidney transplant patients, in which there is evidence that adherence to immunosuppressants increased among participants due to a health education program about dosages, frequency of intake, adverse effects of medications, as well as the effects of non-adherence^(25)^.

In contrast, the findings demonstrate that there are different perceptions about the object of adherence to treatment after transplantation on the part of people undergoing transplantation and professionals involved in care, as different categories were observed, in particular those mentioned by patients related to disease prevention and care of the surgical wound.

Moreover, post-transplant dietary care appears to be more valued by patients than by professionals involved in transplantation, which is possibly related to restrictions applied to the terminal chronic disease experienced by these people before the procedure. In addition, the start or intensification of the use of insulin, a subcutaneous medication, known to be more complex to administer, is another factor that seems to be perceived as something of greater importance by patients than by professionals.

Given the above, it is clear that health care becomes even more important to ensure the success of the procedure and the recipient’s quality of life. In addition to essential immunosuppressive therapy, attention to all of the individual’s organic systems, as well as daily lifestyle habits, play a fundamental role in the recovery and adaptation process in the post-transplant context.

The study has limitations. One of them may be related to the performance of the FG with patients in a single transplant center and the fact that the participants do not faithfully represent the needs and perceptions of care in the post-renal transplant period. The lack of homogeneity among the professionals in the study is also a limitation, since most of the results demonstrated post-transplant care by doctors and nurses, while the distinction of other categories present in the transplant team was deficient. However, even with the limitations, the findings presented here can be adopted in the development of care protocols for people undergoing kidney transplantation, as well as in the development of educational materials aimed at this audience.

### Advances in Healthcare

Since this is an alternative treatment for CKD that requires continuous monitoring by professionals until possible graft loss and/or death, the study was essential for the joint development, by transplant recipients and the healthcare team, of a healthcare handbook, with the aim of improving health monitoring with the multidisciplinary team after kidney transplantation through more effective communication of clinical aspects within healthcare levels, and educational communication, providing important information regarding self-care, which can contribute to better adherence rates to transplant treatment, as well as prevent infections and kidney graft loss, actions that are so important for better survival rates and quality of life.

## CONCLUSIONS

Given the results found, we can infer that patients’ major concern is related to the use of medications, especially immunosuppressants, and eating habits. However, there is also an understanding of the importance of information provided at hospital discharge related to therapeutic follow-up and lifestyle habits after kidney transplantation.

Conversely, healthcare professionals consider the use of medication, health monitoring through laboratory tests, and attendance at consultations to be extremely important for the proper functioning of the transplanted graft. In addition, they also highlight the importance of non-pharmacological care to prevent infections and achieve a better quality of life post-transplant.
