# Supplementary material for: Perception of transplant recipients and professionals about health care following kidney transplantation
Source: Rev Esc Enferm USP. 2025 Feb 14;58:e20240237. doi: 10.1590/1980-220X-REEUSP-2024-0237en (PMC11835224; doi:10.1590/1980-220X-REEUSP-2024-0237en)
Supplement: Translation

# Translation: Percepção de pessoas transplantadas e dos profissionais acerca dos cuidados de saúde após o transplante renal

**DOI:** 10.1590/1980-220X-REEUSP-2024-0237pt

**Authors:** Sâmia Jucá Pinheiro, Carla Daniara Feitosa Coelho, Jean Augusto da Silva, Cristefânia Meirú de Lima, Annaíza Freitas Lopes de Araújo, Klarissa Karine Lima Maracaipe, Dayani Galato

**Affiliations:** 1 Empresa Brasileira de Serviços Hospitalares, Hospital Universitário Walter Cantídio, Fortaleza, CE, Brazil.; 2 Universidade de Brasília, Faculdade de Ceilândia, Brasília, DF, Brazil.; 3 Empresa Brasileira de Serviços Hospitalares, Hospital Universitário de Brasília, Brasília, DF, Brazil.

**Keywords:** Transplante de Rim, Cuidados Pós-Operatórios, Assistência Integral à Saúde, Comunicação em Saúde

## Abstract

**Objetivo:**

Explorar as percepções das pessoas transplantadas renais e dos profissionais acerca dos cuidados de saúde após o transplante.

**Método:**

Estudo exploratório, qualitativo, realizado por meio de grupos focais e aplicação de questionário, em 2023, com pessoas transplantadas renais e profissionais de saúde. A análise foi realizada com o apoio do software IRaMuTeQ^®^.

**Resultados:**

Participaram 24 pessoas transplantadas e 18 profissionais. A partir do processamento dos dados, emergiram dois grupos temáticos: uso de medicamentos imunossupressores e hábitos alimentares no pós-transplante renal; e outros cuidados não-farmacológicos e monitoramento de saúde no pós-transplante.

**Conclusão:**

A maior preocupação das pessoas transplantadas está relacionada ao uso dos medicamentos e aos hábitos alimentares; e os profissionais consideram o uso de medicamentos e o monitoramento de saúde extremamente importantes para um bom funcionamento do enxerto transplantado. Contudo, há uma importância dos cuidados não-farmacológicos, para os dois grupos, diante de um adequado seguimento terapêutico.

## INTRODUÇÃO

O transplante renal é considerado uma terapia renal substitutiva para pacientes com doença renal crônica (DRC) avançada, oferecendo uma melhor sobrevida em relação à hemodiálise(^(1)^. No entanto, o sucesso em longo prazo depende não apenas dos procedimentos médicos-cirúrgicos, mas também da qualidade dos cuidados recebidos antes e após a realização desses procedimentos(^(2)^. A forma como as pessoas transplantadas e seus familiares percebem e se envolvem com esses cuidados pode impactar significativamente na adesão ao tratamento, e consequentemente nos desfechos, sejam eles clínicos ou mesmo humanísticos^(3)^.

Os indivíduos que realizaram um transplante de rim enfrentam um itinerário complexo desde o pré-transplante, que envolve a realização de vários exames e consultas a fim de torná-los aptos para entrar em lista de espera pelo órgão. Após o procedimento, eles devem aderir rigorosamente a um regime de medicamentos imunossupressores, realizar acompanhamentos regulares com a equipe de saúde, bem como adotar mudanças no estilo de vida com o intuito de prevenir infecções e minimizar os riscos de rejeição ao órgão transplantado.

Essa carga de cuidados contínuos pode ser desafiadora para os pacientes, afetando o bem-estar físico e emocional, mas também para os profissionais e instituições de saúde diante da logística a ser executada e os custos enfrentados para alcançar o objetivo^(4)^.

Os profissionais de saúde também desempenham um papel fundamental na prestação de cuidados no pré e pós-transplante renal. O conhecimento técnico-científico sobre os protocolos de tratamento, sobre as dificuldades enfrentadas por essas pessoas diante da complexidade na gestão do autocuidado, e acerca das estratégias para melhorar a adesão, é essencial para moldar as práticas de cuidado. No entanto, esses profissionais também enfrentam desafios frequentemente relacionados à carga de trabalho elevada e à habilidade reduzida em compreender as necessidades de cada paciente transplantado, fundamental para promover uma assistência personalizada^(5)^.

O vínculo e a colaboração mútua entre profissionais de saúde e pacientes diante desses multicomponentes terapêuticos configuram-se como componentes vitais para o sucesso em longo prazo dos transplantados renais, onde a importância de explorar as perspectivas e experiências mútuas no contexto dos cuidados pós-transplante ganha um destaque considerável^(6)^.

Diante da complexidade de cuidados após o transplante, acredita-se que qualificar a percepção das pessoas transplantadas renais e dos profissionais de saúde sobre esses cuidados pode desempenhar um papel crucial na gestão efetiva da saúde e no autocuidado com o intuito de melhorar os resultados clínicos e a experiência dessas pessoas por meio de estratégias de adaptação para uma melhor adesão ao tratamento.

Diante do exposto, o estudo apresenta como objetivo explorar as percepções das pessoas transplantadas renais e dos profissionais acerca dos cuidados de saúde após o transplante.

## METODOLOGIA

### Tipo de Estudo

Trata-se de um estudo exploratório, descritivo, apresentando uma abordagem qualitativa por meio de Grupo Focal (GF) e aplicação de questionários.

### Local, População e Critérios de Seleção

O estudo foi realizado no ambulatório de transplante renal, em agosto de 2023, com 24 pessoas submetidas ao transplante renal e 18 profissionais de saúde atuantes na unidade de transplante, no período de agosto a dezembro de 2023, todos em um hospital de referência do Distrito Federal.

### Definição da Amostra

Foram incluídas pessoas transplantadas renais atendidas no ambulatório de pós-transplante com idade mínima de 18 anos, e excluídos os que apresentaram estado de letargia na ocasião da coleta. As pessoas foram convidadas a participar da pesquisa enquanto aguardavam o atendimento no ambulatório.

Os profissionais de saúde incluídos no estudo apresentaram os seguintes critérios: atuação na unidade de transplante há, pelo menos, um ano, e que tivessem especialização e/ou residência em transplante, independente do tempo de atuação. O critério de exclusão foi estar de férias ou licença.

### Coleta de Dados

Foram realizados dois GF, com12 participantes, com as pessoas transplantadas: por meio de um roteiro específico com perguntas e aspectos referentes às orientações de cuidados de saúde após o transplante renal e questões de interesse sugeridas pelos participantes durante a realização dos grupos, em dias distintos, que duraram aproximadamente 90 minutos cada. Os participantes foram convidados enquanto esperavam a consulta no ambulatório de transplante renal nos referidos dias de realização dos grupos. Todos que estavam na sala de espera aceitaram o convite e nenhum desistiu da participação no decorrer da atividade. O grupo foi conduzido por um profissional e contou com três relatores. Os encontros foram gravados em áudio.

As informações referentes aos profissionais de saúde foram coletadas por meio da aplicação de um questionário elaborado pelos autores e aplicado de forma física, contendo questões acerca da categoria profissional, titulação e tempo de serviço e uma pergunta aberta acerca dos cuidados indispensáveis aos pacientes após o transplante renal.

### Análise e Tratamento dos Dados

Os dados sociodemográficos e clínicos dos pacientes e as questões profissionais, foram submetidas à análise descritiva. Os dados oriundos dos aspectos abordados em torno da temática no GF, obtidos por meio da transcrição dos áudios e das anotações da relatoria, e os originados das respostas dos profissionais foram inseridos em um banco de dados e processados no *software Interface de R pour Analyses Multidimensionnelles de Textes et de Questionneires* (IRaMuTeQ^®^), versão 0.7 Alpha2, sendo realizada uma análise lexical por meio da Análise de Similitude e Classificação Hierárquica Descendente.

O IRaMuTeQ^®^ combina métodos estatísticos avançados, tornando-se acessível a pesquisadores com diversos níveis de experiência em análise de dados. A análise de similitude é uma técnica para a análise de dados textuais, proporcionando uma compreensão visual e semântica do conteúdo textual, e permite que os pesquisadores explorem a estrutura e o conteúdo dos textos de forma abrangente^(7,8)^. A Classificação Hierárquica Descendente é uma técnica que permite agrupar elementos textuais semelhantes para identificar estruturas semânticas em grandes volumes de texto. No IRaMuTeQ^®^, o teste qui-quadrado (X^2^) é uma ferramenta estatística utilizada para analisar a associação entre palavras e categorias em um *corpus* de texto, auxiliando na construção de um dendrograma que representa as classes com suas palavras mais características^(9)^.

### Aspectos Éticos

O estudo foi decorrente da realização de duas das etapas de pesquisa de doutorado e ocorreu após analisado e aprovado pelo Comitê de Ética em Pesquisa da Faculdade de Ceilândia, sob parecer nº 67087723.2.0000.8093, conforme a Resolução nº 466, de 12 de dezembro de 2012, do Conselho Nacional de Ética em Pesquisa. Os participantes foram informados que a participação era voluntária e foi destacado que a recusa não acarretaria nenhum prejuízo assistencial para os pacientes, como também não teria contrariedade dos pesquisadores em relação à provável recusa dos profissionais. Todos assinaram o termo de consentimento previamente.

Para preservar o anonimato, os pacientes foram caracterizados pela letra P e seu número de identificação, seguido da letra G e o número referente à ordem de realização do GF, como por exemplo: P1G1 (paciente um do primeiro GF realizado). Os profissionais foram caracterizados pela letra E e seu número de identificação, como por exemplo E1 (profissional um). A elaboração do estudo seguiu as recomendações para a elaboração de pesquisas qualitativas do COREQ (Critérios Consolidados para Relatar uma Pesquisa Qualitativa).

## RESULTADOS

Participaram do estudo 24 pessoas transplantadas e 18 profissionais de saúde. Inicialmente, serão apresentados os dados de caracterização das pessoas submetidas ao transplante (Tabela 1).

**Tabela 1:** erfil sociodemográfico e clínico das pessoas transplantadas que participaram dos GF sobre a percepção do cuidado após o transplante renal – Brasília, DF, Brasil, 2023.

Pacientes transplantados renais	N	%
**Sexo**		
Feminino	12	50
Masculino	12	50
**Raça**		
Negra	1	4%
Parda	21	88%
Amarela	0	0
Branca	2	8%
**Estado civil**		
Solteiro	3	12%
Casado	5	21%
União estável	11	46%
Divorciado	4	17%
Viúvo	1	4%
**Escolaridade**		
Sem escolaridade	1	4%
Ensino fundamental	8	33,5%
Ensino médio	7	29,5%
Ensino Superior	6	25%
Pós-graduação	2	8%
**Idade**		
Adultos	16	66,5%
Idosos	8	33,5%
**Causa DRC**		
Desconhecida	16	66,5%
Doença renal policística autossômica dominante	3	12,5%
Diabetes Mellitus	2	8%
Nefropatia por IgA	1	4%
Glomerulonefrite crescêntica	1	4%
Nefrotoxicidade por antibioticoterapia	1	4%
**Tempo de transplante (anos)**		
<1	10	41,5%
1–5	9	37,5%
Acima de 5	5	21%
**Tipo de doador**		
Vivo	5	21%
Falecido	19	79%
**Retransplante**		
Sim	4	17%
Não	20	83%
**Total**	**24**	**100%**

As pessoas transplantadas estavam distribuídas de forma homogênea em relação ao sexo. Majoritariamente se consideravam pardos; a maioria era casada ou apresentava união estável, correspondendo a 67%. Em relação à idade, 66,5% eram adultos e 33,5% idosos. A maioria desconhecia a causa da DRC, correspondendo a 66,5%.

Os profissionais participantes apresentaram categorias diversas, estando representados em maior número por médicos e enfermeiros, ambos com quatro e oito, respectivamente; dois fisioterapeutas; um farmacêutico; um nutricionista; um assistente social; e um psicólogo. A maioria (13) tinha o título de especialista ou residência; quatro eram mestres e um tinha doutorado. Nove apresentavam menos de cinco anos no serviço de transplante renal, e apenas um apresentava mais de dez anos de atuação.

A análise de similitude dos textos obtidos por meio dos GF com as pessoas transplantadas e dos questionários com os profissionais de saúde que atuavam na assistência permitiu a elaboração da Figura 1. Nas imagens, é possível observar a síntese das palavras que permitem a compreensão do objeto do estudo.

**Figura 1:** Análise de similitude correspondente aos resultados dos grupos focais com os pacientes e dos questionários com os profissionais sobre os cuidados de saúde no pós-transplante renal.

Entre as palavras que se destacaram nos GF, observaram-se quatro eixos organizados de forma transversal. São eles: *transplante, saber, importante e informação*. A palavra *orientação* aparece como uma ramificação no eixo do *transplante*, que está ligada diretamente ao eixo do *saber*. No eixo das palavras *informação* e *importante* aparecem ramificações relacionadas à *alimentação* e aos *medicamentos*; e uma ramificação mais horizontal relacionada à palavra *tomar*, estando ligada ao uso de *medicamentos* e *vacinas*.

A representação gráfica das palavras evocadas pelos pacientes demonstra a compreensão relacionada ao que é importante após o transplante, bem como as necessidades de orientação e informação diante do seguimento terapêutico, em especial aos aspectos relacionados aos medicamentos e alimentação, como pode ser evidenciado pela transcrição das falas de alguns participantes:

P1G1: *No começo achei que ia morrer de tanto medicamento que deveria usar e não entendia nada. Me disseram que eu não era pra comer muita carne vermelha. Fico perdida sem saber o que comer.*

P2G1: *Quando tive alta, a nutricionista deu orientação geral, algo muito grosseiro. A questão da alimentação, o que serve para mim pode não servir para o outro. Quando saem os resultados dos exames ficamos sem saber o que comer. Outra paciente quando teve alta junto comigo não sabia o que comer.*

P3G1: *Seria interessante ter orientações em relação à aplicação de insulina, tive muito medo de aplicar sozinha quando comecei a usar, 4 anos depois do transplante. Se tivesse vídeo, figura... explicando como aplicar, teria sido melhor.*

P9G1: *A dieta gera dúvida, na alta entregam muito superficialmente. É importante a relação da alimentação com os resultados dos exames. Eu já percebo quando como algo errado, já percebo mudança no meu corpo... fico tonta, dor de cabeça.... e quando vem o resultado dos exames eu já sei que extrapolei ali.*

P1G2: *A principal informação é da alimentação, tem que ter o que pode ou não comer para não perder o rim. Na hemodiálise sabíamos o que fazer. Os medicamentos também são bem importantes... medicamento certo e na hora certa porque senão tem rejeição.*

P2G2: *Informações de como tomar os medicamentos.... horários, doses... achei ótimo receber a tabela dos medicamentos na alta explicando direitinho como usar a prednisona.*

P3G2: *Cuidado maior que tem que ter é com alimentação e água. Não bebo água de qualquer lugar. Na hemodiálise, se abusar da comida, a máquina tira... com o transplante não tem isso.*

P4G2: *Alimentação, medicamento e atividade física são as informações mais importantes que eu acho que deve ter.*

Já com os dados referentes à equipe multiprofissional, a palavra *paciente* aparece mais centralizada diante do *corpus* textual. Dela se ramificam as palavras *transplante* e *cuidado* de forma mais estreita, e a palavra *imunossupressor* se caracteriza de forma evidente, seguida da palavra *evitar* que aparece em uma ramificação mais abaixo. *Equipe, acompanhamento, tratamento, informação,* e *complicação* aparecem mais atreladas à palavra *paciente*; e *horário, dosagem, orientação, importância,* e *infecção*, por exemplo, aparecem mais relacionadas à palavra *imunossupressor*. A representação gráfica também evidencia uma preocupação dos profissionais de saúde acerca do que o paciente deve evitar para um melhor desenvolvimento do tratamento, onde se percebe a relação da palavra *evitar* com *alimentação, exercício, atividade* e *contato*.

O destaque da palavra *imunossupressor* e *orientação* fica bastante evidente nos relatos dos participantes, em que a maioria destacou o uso correto dos medicamentos como a primeira orientação a ser fornecida no pós-transplante, podendo ser observado em vários relatos, estando representados alguns deles:

E2: *As principais orientações são: Tomar os medicamentos nos horários corretos, não esquecer de nenhuma dose; tomar os imunossupressores longe das refeições.*

E3: *Algumas das medidas indispensáveis incluem: Cuidados com os imunossupressores - adesão rigorosa à terapia imunossupressora. Para prevenir a rejeição do órgão transplantado o paciente deve tomar os remédios conforme a prescrição médica, respeitando os horários e as doses indicadas.*

E10: *Durante a alta hospitalar, os pacientes precisam ser orientados quanto aos riscos de perda do transplante oriundos do uso inadequado dos imunossupressores.*

Apesar da evidência marcante em torno da imunossupressão, também há uma preocupação com os cuidados não-farmacológicos, em que se destaca uma ligação importante na demonstração gráfica entre *paciente* e *cuidado*, inseridas na mesma zona de *transplante,* e com ramificação importante com as palavras *informação*, *acompanhamento* e *tratamento*, o que se pode destacar por meio de alguns dos relatos:

E1: *Cuidados indispensáveis no pós-operatório tardio – após três ou quatro meses da cirurgia, é permitido realizar exercícios físicos em academia, como forma de treinamento e condicionamento físico. Para isso deve conversar com o seu médico e solicitar a liberação. Vários benefícios são observados com a prática de atividade física regular, dentre eles: prevenção de osteoporose, diabetes e hipertensão; ganho de massa muscular; melhora na qualidade do sono e humor.*

E12: *Adesão ao tratamento medicamentoso e não medicamentoso também (hábitos de vida saudáveis para o êxito no tratamento - alimentação, atividade física e assiduidade no acompanhamento com a equipe de saúde).*

E13: *Uso de máscaras, protetor solar. Evitar aglomerações, principalmente nos seis primeiros meses após o transplante. Higienizar as mãos antes das refeições e após o uso do banheiro. Frequentar as consultas e realizar os exames regularmente.*

E18: *Acompanhamento de saúde com o olhar nos aspectos emocionais dos pacientes.*

Na Classificação Hierárquica Descendente (Método de Reinert), por meio do processamento dos dados oriundos do GF com os pacientes, foram analisados 111 segmentos de texto (ST) com um aproveitamento de 80,18% dos ST, o que gerou quatro classes que foram denominadas por meio da ideia transmitida pelas palavras encontradas. São elas – Classe 1 – Cuidados pós-operatórios; Classe 2 – Uso de medicamentos e alimentação no pós-transplante renal; Classe 3 – Prevenção de doenças; e Classe 4 – Acompanhamento com a equipe de saúde. Na Figura 2, é apresentado o dendrograma com a relação entre as classes.

**Figura 2:** Dendrograma correspondente às classes encontradas por meio do processamento do *corpus* textual oriundo dos GF realizados com as pessoas transplantadas.

Observa-se que a classe 1 representou 23,6% dos segmentos de texto; as classes 2 e 3 representaram 28,1% cada uma; e a 4 representou 20,2%. Foram incluídos 10 vocábulos em cada classe, sendo o ponto de corte para a inclusão o valor do qui-quadrado (X^2^) maior ou igual a 3,84 e com p < 0,05, sendo apresentados no dendrograma a fim de propiciar uma melhor compreensão diante da elaboração das classes.

As classes geradas a partir da Classificação Hierárquica Descendente por meio do processamento dos dados obtidos pelos questionários com os profissionais de saúde, e com um aproveitamento de 90,67% dos ST, foram denominadas de: Classe 1 – Acompanhamento com a equipe de saúde; Classe 2 – Uso responsável de medicamentos; Classe 3 – Cuidados não-farmacológicos no pós-transplante; Classe 4 – Monitoramento de saúde no pós-transplante renal.

Demonstra-se, na Figura 3, as classes encontradas por meio do processamento dos dados provenientes dos questionários da equipe multiprofissional.

**Figura 3:** Dendrograma correspondente às classes encontradas por meio do processamento do *corpus* textual dos questionários aplicados aos profissionais da saúde que atuam na assistência à pessoa transplantada renal.

A classe 1 representou 25% dos ST; a 2, 17,6%; a classe 3 representou 29,4%; e a 4, representou 27,9%. Assim como no dendrograma relacionado ao *corpus* textual dos pacientes, os vocábulos, que também tiveram como ponto de corte o valor do qui-quadrado (X^2^) maior ou igual a 3,84, e com p < 0,05, foram apresentados no dendrograma dos profissionais a fim de propiciar uma melhor compreensão diante da elaboração das classes.

As classes geradas por meio dos vocábulos mais frequentes no *corpus* textual, nos dois grupos analisados, foram extremamente válidas para nortear a discussão dos achados do presente estudo com outras pesquisas publicadas, destacando as necessidades de cuidados mais prevalentes nos pacientes, como também enfatizando o direcionamento do cuidado dos profissionais no pós-transplante.

## DISCUSSÃO

Na percepção das pessoas transplantadas, a informação relacionada à alimentação e ao uso de medicamentos apresentou-se de forma evidente, bem como a importância da realização dos exames e comparecimento nas consultas, além de outras ramificações relacionadas aos cuidados com a ferida operatória, prevenção de doenças, cuidado com o domicílio, e contato com animais. Já na percepção dos profissionais de saúde vinculados à assistência, o paciente foi inserido no centro do cuidado e foi demonstrada uma maior importância dos cuidados farmacológicos, relacionados principalmente aos medicamentos imunossupressores. Contudo, também há o destaque para os cuidados não-farmacológicos, demonstrando, por meio da análise lexical, a importância da prática de alimentação e atividade física para uma melhor qualidade de vida após o transplante.

Neste contexto, por meio da análise de similitude e método de Reinert, foram criados dois grupos temáticos norteadores da discussão dos dados. São eles:

### Uso de Medicamentos Imunossupressores e Hábitos Alimentares no Pós-Transplante Renal

A importância direcionada aos medicamentos pelas pessoas transplantadas e profissionais é compreensível, tendo em vista que a administração segura dos imunossupressores é fundamental, uma vez que esses fármacos desempenham um papel na prevenção da rejeição do órgão transplantado ao suprimir a resposta imunológica do indivíduo. O sucesso do transplante depende da adesão a um regime de medicamentos prescritos, seguindo rigorosamente as doses e os horários recomendados pela equipe de saúde^(10)^.

Entre os mais comuns estão tacrolimo, everolimo, sirolimo, micofenolato sódico e mofetila, ciclosporina, azatioprina e corticosteroides. Esses fármacos podem apresentar interações com outros medicamentos e alimentos, bem como propiciar eventos adversos que podem impactar a qualidade de vida dos pacientes^(11)^. Dessa forma, é essencial fornecer informações detalhadas aos pacientes sobre o uso correto dos medicamentos, bem como os diversos problemas que podem surgir, como o aumento do risco de infecções, diabetes, hipertensão, dislipidemias e câncer^(12,13)^.

No estudo atual, há relatos dos pacientes que demonstraram uma preocupação em relação às restrições alimentares, principalmente nos que faziam a hemodiálise ou diálise peritoneal como terapias substitutivas antes do transplante. O fato é que as pessoas transplantadas renais passam a apresentar recomendações nutricionais distintas em virtude do funcionamento adequado do novo rim. No entanto, muitos pacientes ainda ficam receosos em relação ao consumo de alimentos antes considerados proibidos ou não recomendados durantes as outras terapias renais substitutivas^(14)^.

Recomenda-se uma dieta balanceada, rica em frutas, vegetais, grãos integrais e proteínas magras, enquanto se limita o consumo de sódio, gorduras saturadas e açúcares^(15)^. A avaliação dos níveis séricos de sódio, potássio, fósforo e magnésio, por exemplo, é importante para definir conduta entre a restrição ou suplementação caso o paciente necessite. A ingestão adequada de líquidos também é importante para manter a hidratação e a função renal adequada. É fundamental uma adequada higiene alimentar, que inclui lavar bem os alimentos, refrigerar alimentos perecíveis e evitar o consumo de alimentos potencialmente contaminados e/ou preparados em locais de procedência desconhecida ou duvidosa^(14)^.

Alguns alimentos podem interagir com os medicamentos, alterando a sua eficácia ou aumentando o risco de efeitos colaterais. Por exemplo, o consumo excessivo de toranja pode aumentar os níveis sanguíneos de certos medicamentos, como o tacrolimo e a ciclosporina; assim como suplementos à base de plantas podem interferir na função renal e/ou diminuir a eficácia dos imunossupressores. Portanto, é fundamental o seguimento de orientações específicas sobre alimentação pelo nutricionista desde a alta hospitalar^(13)^.

Em suma, a dieta da pessoa transplantada renal deve ser planejada de forma cuidadosa para evitar interações com medicamentos, principalmente com os imunossupressores, bem como para promover uma saúde adequada no longo prazo, diminuindo os riscos de desenvolvimento de dislipidemias, diabetes, e hipertensão arterial, por exemplo^(16)^.

### Outros Cuidados Não-Farmacológicos e Monitoramento de Saúde no Pós-Transplante

Além da terapia medicamentosa, os pacientes necessitam de cuidados gerais com o intuito de apresentar um bom estado de saúde ao longo do tempo. Para isso, o cuidado com as pessoas transplantadas deve ser holístico e integral. Isto inclui cuidados para a adoção de hábitos de vida saudáveis, como a realização de atividade física, prática sexual segura, e gestão do estresse, bem como práticas referentes à prevenção do desenvolvimento de doenças crônicas e do acometimento de doenças infectocontagiosas, sendo fundamental o comparecimento às consultas e exames com a equipe multiprofissional^(17)^.

A prática sexual é liberada a partir de seis semanas após o transplante ou até que a pessoa esteja se sentindo disposta e não apresente dores. No entanto, as pessoas devem evitar relações sexuais desprotegidas para reduzir o risco de infecções que poderiam afetar negativamente o rim transplantado ou causar complicações graves. É aconselhável comunicar quaisquer dúvidas relacionadas à atividade sexual à equipe de saúde responsável pelo acompanhamento, pois eles podem oferecer orientações personalizadas para o alcance de uma vida sexual saudável e segura. Além disso, é importante as mulheres transplantadas comunicarem o desejo de engravidar, uma vez que a gestação é recomendada somente um ano após o transplante e, muitas vezes, há uma modificação nos medicamentos imunossupressores^(17)^.

O estudo atual, por meio dos resultados encontrados, evidencia a importância da detecção precoce e prevenção de doenças entre as pessoas transplantadas de rim. Elas devem ser orientadas quanto à presença de sinais flogísticos na região da ferida operatória, febre persistente, calafrios, dor ao urinar, aumento da dor ou sensibilidade na região do rim transplantado, fadiga intensa, mal-estar e alterações no apetite. Esses sinais podem indicar desde infecções locais na ferida operatória e no trato urinário, até infecções sistêmicas graves. A identificação e o tratamento dessas intercorrências, de forma rápida, podem preservar a função do rim transplantado e a qualidade de vida do indivíduo^(18)^.

Outro aspecto de cuidado após o transplante está relacionado à orientação quanto à imunização das pessoas transplantadas desde antes do transplante, sendo crucial o recebimento de todas as vacinas recomendadas, uma vez que, após o transplante, vacinas compostas por vírus vivo e/ou atenuado não podem ser administradas devido ao risco de ocasionar uma infecção ativa em virtude da condição de supressão imunológica das pessoas transplantadas^(19)^.

A imunização passiva nas pessoas transplantadas é menos eficaz do que nos indivíduos imunocompetentes, razão pela qual se torna imprescindível a vacinação das pessoas que receberam transplante, principalmente devido a uma maior vulnerabilidade às infecções. A hesitação individual em relação à vacinação, muitas vezes promovida por movimentos antivacina, pode colocar em risco a saúde das pessoas, diminuindo a proteção da comunidade contra doenças evitáveis por vacinação. Portanto, é fundamental que os profissionais de saúde informem os benefícios das vacinas para a população em geral, bem como orientem quanto aos malefícios gerados pela não vacinação^(20,21)^.

É essencial, também, que as pessoas transplantadas sejam instruídas acerca da prevenção de picadas de mosquitos, bem como em relação à busca imediata por atendimento médico se apresentarem sintomas sugestivos de arboviroses, como dengue, zica e chikunguya, para um manejo precoce que minimize o risco de complicações graves. Embora o curso da doença pareça semelhante com a população em geral, pessoas transplantadas renais que desenvolvem a dengue, por exemplo, podem apresentar maiores complicações devido à resposta imunológica comprometida e presença de comorbidades associadas, o que resultaria em um maior tempo de hospitalização^(22)^.

Um estudo publicado em 2023 acerca de um *Benchmarking* das tecnologias educativas em saúde para auxiliar na adesão terapêutica dos pacientes transplantados destacou que as orientações relacionadas à importância da realização de exames e retorno às consultas, aos aspectos relacionados à higiene e acerca do manejo nas intercorrências clínicas pós-transplante estavam presentes em todas as tecnologias encontradas, juntamente com uso responsável de medicamentos e prática alimentar saudável^(23)^. Prática de atividade física também foi evidenciada na maioria das tecnologias disponíveis. Diante disso, as necessidades dos pacientes e percepções de cuidados dos profissionais destacadas neste estudo corroboram a pesquisa citada.

A atividade física torna-se fundamental no pós-transplante renal devido aos benefícios que proporciona, como: melhorar a saúde cardiovascular; manter um peso adequado reduzindo o risco de complicações metabólicas; e promover o fortalecimento muscular. Um estudo desenvolvido em 2023, nos Estados Unidos, sobre o desenvolvimento de um programa de estilo de vida entre os transplantados renais, destaca que a participação dos pacientes foi fundamental para desenvolver comportamentos saudáveis no programa, e revelou que os transplantados apresentaram interesse em aderir ao programa com treinamento de atividade física e personalização nutricional^(24)^.

Para auxiliar melhor na adesão ao tratamento os pacientes necessitam de uma educação contínua no tratamento de transplante. Dessa forma, os profissionais precisam de constante aperfeiçoamento e realização de práticas educativas com o intuito de auxiliar nesse processo e, consequentemente, melhorar a sobrevida e qualidade de vida. Isso pode ser demonstrado por meio de um estudo realizado em um hospital de grande porte na Etiópia com pacientes transplantados renais, em que há evidências de que a adesão aos imunossupressores aumentou entre os participantes em virtude de um programa de educação em saúde acerca das dosagens, frequência de ingestão, efeitos adversos dos medicamentos, bem como os efeitos da não adesão^(25)^.

Por outro lado, os achados demonstram que há diferentes percepções sobre o objeto adesão ao tratamento após o transplante por parte das pessoas submetidas ao transplante e dos profissionais envolvidos na assistência, pois observaram-se diferentes categorias, em especial destaca-se aquelas citadas pelos pacientes relacionadas à prevenção de doenças e aos cuidados com a ferida cirúrgica.

Além disso, os cuidados alimentares pós-transplante parecem ser mais valorizados pelos pacientes do que pelos profissionais que atuam no transplante, o que possivelmente guarda relação com as restrições relacionadas ao quadro de doença crônica em fase terminal vivenciada por essas pessoas antes do procedimento. Ainda, o início ou a intensificação do uso da insulina, medicamento subcutâneo, sabidamente com administração com maior complexidade, é outro fator que parece ser percebido como algo de maior importância pelos pacientes do que pelos profissionais.

Diante do exposto, observa-se que o cuidado com a saúde se torna ainda mais importante para garantir o sucesso do procedimento e a qualidade de vida do receptor. Além da imprescindível terapia imunossupressora, a atenção a todos os sistemas orgânicos do indivíduo, bem como aos hábitos de vida diários, desempenha um papel fundamental no processo de recuperação e adaptação no contexto pós-transplante.

O estudo apresenta limitações. Uma delas pode estar relacionada à realização do GF com os pacientes em um único centro transplantador e ao fato de os participantes não representarem fielmente as necessidades e percepções dos cuidados no pós-transplante renal. A não homogeneidade entre os profissionais do estudo também se configura em uma limitação, uma vez que a maioria dos resultados demonstrou os cuidados pós-transplante por médicos e enfermeiros, ficando deficiente o destaque entre as outras categorias presentes na equipe do transplante. No entanto, mesmo com as limitações, os achados aqui apresentados podem ser adotados no desenvolvimento de protocolos de cuidado às pessoas submetidas ao transplante renal, bem como ao desenvolvimento de materiais educativos voltados a esse público.

### Avanços Para a Área da Saúde

Em se tratando de uma alternativa de tratamento para a DRC e que necessita de um acompanhamento com os profissionais de forma contínua até uma possível perda do enxerto e/ou óbito, o estudo foi fundamental para o desenvolvimento conjunto, por pessoas transplantadas e equipe de saúde, de uma caderneta de saúde assistencial, com o intuito de melhorar o acompanhamento de saúde com a equipe multiprofissional após o transplante renal por meio de uma comunicação mais efetiva dos aspectos clínicos entre os níveis de atenção à saúde, e educativa, dispondo de informações importantes referentes ao autocuidado, o que pode contribuir para melhores índices de adesão ao tratamento de transplante, bem como prevenir infecções e a perda do enxerto renal, ações tão importantes para melhores índices de sobrevida e qualidade de vida.

## CONCLUSÕES

Diante dos resultados encontrados, podemos inferir que a maior preocupação dos pacientes está relacionada ao uso dos medicamentos, principalmente imunossupressores, e aos hábitos alimentares. Contudo, também há uma compreensão da importância das informações dispensadas na alta hospitalar relacionadas ao seguimento terapêutico e aos hábitos de vida após o transplante renal.

Por outro lado, os profissionais da equipe de saúde consideram o uso de medicamentos, o monitoramento de saúde por meio da realização de exames laboratoriais e o comparecimento às consultas extremamente importantes para um bom funcionamento do enxerto transplantado. De forma complementar, destacam, ainda, a importância dos cuidados não-farmacológicos para prevenir infecções e alcançarem uma melhor qualidade de vida no pós-transplante.
